# Presenting Symptoms and Stage at Diagnosis Among Women Diagnosed With Breast Cancer Through a Combined Breast Cancer and Cervical Cancer Screening Program in Rwanda

**DOI:** 10.1200/GO.22.56000

**Published:** 2022-05-05

**Authors:** Lydia Pace, Jean-Marie Vianney Dusengimana, Jean Paul Balinda, Marie Origene Benewe, Vestine Rugema, Jean de Dieu Uwihaye, Amanda Fata, Cyprien Shyirambere, Lawrence Shulman, Nancy Keating, Francois Uwinkindi, Marc Hagenimana

**Affiliations:** ^1^ Brigham and Women's Hospital, Boston, MA; ^2^ Partners in Health, Boston, MA; ^3^ Rwanda Biomedical Centre, Kigali, Rwanda; ^4^ Rwanda Ministry of Health, Kigali, Rwanda; ^5^ University of Pennsylvania, Philadelphia, PA; ^6^ Harvard Medical School, Boston, MA; ^7^ Rwanda Biomedical Center, Kigali, Rwanda

## Abstract

There is interest in leveraging cervical cancer (CC) screening to facilitate early breast cancer (BC) detection in low- and middle-income countries (LMIC). We sought to determine whether adding clinical breast exams (CBE) to CC screening led to early BC diagnoses among asymptomatic women in Rwanda's Women's Cancer Early Detection Program (WCEDP). The WCEDP provided CBE to women aged 30-49 who were receiving CC screening, as well as any individual with breast symptoms.

**METHODS:**

The WCEDP was launched in three Rwandan districts (total population 1.3 million) in July 2018, August 2018 and May 2019 respectively. This analysis included patients presenting to health centers (HCs) through December 31, 2019. Follow-up data were collected through April 2021 using clinicians' weekly reports, patient navigator referral data, and the cancer hospital's electronic medical record. We determined patients' initial symptoms from HC records, patient interviews, and phone surveys.

**RESULTS:**

Nine thousand seven hundred sixty-three women received CC screening and CBE together; 7,616 additional women received CBE alone. Five hundred eighty-five women were referred from HCs to a district hospital (DH) for abnormal CBE; 200 were referred from the DH to the referral hospital. Twenty-nine women were diagnosed with BC; of these 19 (66%) were 50 or older and 23 (79%) had stage III/IV disease. Median interval from HC visit to referral hospital visit was 19 days (IQR 11.0-26.0). Among the 23 women with BC for whom we could identify their reason for initial HC presentation, all had sought care for breast symptoms. The remaining six had advanced-stage disease and symptomatic tumors at diagnosis.

**CONCLUSION:**

During the initial rollout of this combined BC and CC screening program, no BC was diagnosed among asymptomatic women and 2/3 women diagnosed with BC were older than the target CC screening age. Adding CBE for all women receiving CC screening in LMIC may be low-yield. Given the high proportion of late-stage diagnoses, community awareness of early BC symptoms, high-quality CBE and timely referrals are important areas of focus.

